# *SNORA13* antisense oligonucleotides enhances the therapeutical effects of 5-fluorouracil in colon adenocarcinoma

**DOI:** 10.3389/fphar.2025.1564682

**Published:** 2025-06-02

**Authors:** Yanzhi Wang, Huihui An, Yaou Zhang, Qing Rex Lyu, Zhe Zhang

**Affiliations:** ^1^ Medical Research Center, Chongqing General Hospital, Chongqing University, Chongqing, China; ^2^ China State Key Laboratory of Chemical Oncogenomics, Tsinghua Shenzhen International Graduate School, Shenzhen, China; ^3^ Key Lab in Healthy Science and Technology of Shenzhen, Tsinghua Shenzhen International Graduate School, Shenzhen, China; ^4^ Department of Chinese Medical Gastroenterology, China-Japan Friendship Hospital, Beijing, China

**Keywords:** *SNORA13*, 5-fluorouracil, colorectal cancer, translation efficiency, nicotinamide N-methyltransferase

## Abstract

**Indroduction:**

Colorectal cancer (CRC) is a prevalent malignancy and is the second leading cause of cancer-related mortality worldwide. 5-Fluorouracil (5-FU) is widely used in clinical intervention, however, drug resistance to 5-FU poses a significant challenge to treatment efficacy. Small nucleolar RNAs (snoRNAs) are a class of nuclear non-coding RNAs that mainly play roles in post-transcriptional RNA processing and modification in ribosomal RNA, which is crucial for sustaining protein synthesis. This study aimed to identify differentially expressed snoRNAs in CRC and pinpoint a specific snoRNA that may exert a synergistic effect with 5-FU administration.

**Methods:**

Combinatorial small RNA array of clinical samples and data analysis from The Cancer Genome Atlas (TCGA) database were used to identify the differentially expressed snoRNAs in colorectal cancer (CRC). To investigate the role of *SNORA13* in CRC, loss-of-function (LoF) study was conducted using transient antisense oligonucleotides (ASOs) transfection and *SNORA13* knockout with CRISPR-Cas9 genome editing in HT29 colon adenocarcinoma cell line. A combined administration of *SNORA13*-ASO and 5-Fluorouracil (5-FU) was performed in nude mice xenograft model to verify the synergistic inhibitory effect. RNA-seq, Ribo-seq and proteomics were performed to identify the downstream target of *SNORA13*, and qRT-PCR and Western Blot were used to confirm the results of multi-omics analysis.

**Results:**

The analysis of small RNA array data combined with the snoRNA expression profile in TCGA database determined that SNORA13 is commonly increased in CRC tissues. The LoF study revealed that the cell proliferation and colony formation are significantly suppressed upon SNORA13 deficiency. Next, the xenografted tumor model in nude mice demonstrated that the smaller tumorigenesis in SNORA13 knockout HT29 cell lines, and SNORA13 ASO enhances the anti-cancer efficacy of 5-FU. Finally, multi-omics analysis and molecular experimental validation revealed that nicotinamide N-methyltransferase (NNMT) is significantly suppressed in SNORA13 knockout HT29 cell lines.

**Conclusion:**

Our study revealed *SNORA13* is highly expressed in CRC and demonstrated knockdown of *SNORA13*, especially combined with 5-FU administration, may represent a promising therapeutic approach for CRC treatment.

## Introduction

Colorectal cancer (CRC) is a prevalent malignancy and is the second leading cause of cancer-related mortality worldwide (Bray et al., 2024). CRC is defined as cancers that develop in the colon or rectum, and colon adenocarcinoma is the most prevalent subtype which arises from glandular epithelial cells and accounts for approximately 90% of all colorectal cancer patients (Dekker et al., 2019; Shin et al., 2023). Up to date, a comprehensive approaches, including surgical operation, chemotherapy, radiation therapy, targeted therapy, and immunotherapy have been applied clinically and gain significant improvement in CRC treatment (Biller and Schrag, 2021). As a classic anti-cancer drug, 5-Fluorouracil (5-FU) is widely used in clinical intervention (Longley et al., 2003). However, the drug resistance of 5-FU is a significant challenge in treatment efficacy (Blondy et al., 2020; Sethy and Kundu, 2021). Therefore, it is urgent need to develop drug that enhances the therapeutical efficacy of 5-Fluorouracil.

Small nucleolar RNAs (snoRNAs) are a class of nuclear non-coding RNA molecules ranging from 60 to 300 nucleotides in length found in eukaryotic cells (Bachellerie et al., 2002). Generally, snoRNAs are classified into two distinct categories: H/ACA box snoRNAs and C/D box snoRNAs. The C/D box snoRNAs, typically ranging from 70 to 120 nucleotides, contain conserved C and D box sequences that facilitate 2′-O-methylation modification (Kiss, 2002). Whereas H/ACA box snoRNAs, spanning 60–75 nucleotides, harbor pseudouridylation pockets crucial for RNA isomerization (McMahon et al., 2015). Beyond these classical functions, recent research has unveiled novel regulatory mechanisms of snoRNAs, including N4-acetylcytidine modification, alternative splicing regulation, and microRNA-like activities (Kishore and Stamm, 2006; Ender et al., 2008; Bortolin-Cavaillé et al., 2022). Initially, these RNA molecules were thought to mainly play roles in post-transcriptional RNA processing and modification in ribosomal RNA, crucial for sustaining protein synthesis (Baßler and Hurt, 2019). However, recent advancements have unveiled that snoRNAs have a much broader and more complex range of functions than previously understood, particularly in the context of cancer development and progression (Williams and Farzaneh, 2012; Huang et al., 2022; Zhang X. et al., 2023).

In this study, our small RNA array data disclosed the upregulation of a serial of snoRNAs in CRC tissues. By combinatorial analysis with the snoRNA expression profile in The Cancer Genome Atlas (TCGA) database, we found *SNORA13* is common increased in CRC tissues. Using antisense oligonucleotides (ASOs) knockdown and generating knockout cell line using CRISPR-Cas9 genome editing, our data revealed that the cell proliferation and colony formation are significantly suppressed upon *SNORA13* deficiency. Next, xenografted tumor model in nude mice was established and our data demonstrated the smaller tumorigenesis in *SNORA13* knockout HT29 cell lines. To study whether suppressing of *SNORA13* exhibits synergistic effect with 5-FU administration, we introduced *SNORA13* ASO and 5-FU respectively and combinatorically, and our data demonstrated *SNORA13* ASO markedly enhances the anti-cancer efficacy of 5-FU. Next, transcriptome and proteome analysis revealed that nicotinamide N-methyltransferase (NNMT) is significantly suppressed in *SNORA13* knockout HT29 cell lines. Together, our data suggested a novel approach of enhancing efficacy of 5-FU by knockdown of *SNORA13*, through suppressing protein synthesis and depleting NNMT.

## Results

### *SNORA13* level is elevated in colorectal cancer tissues and cell lines

**FIGURE 1:**
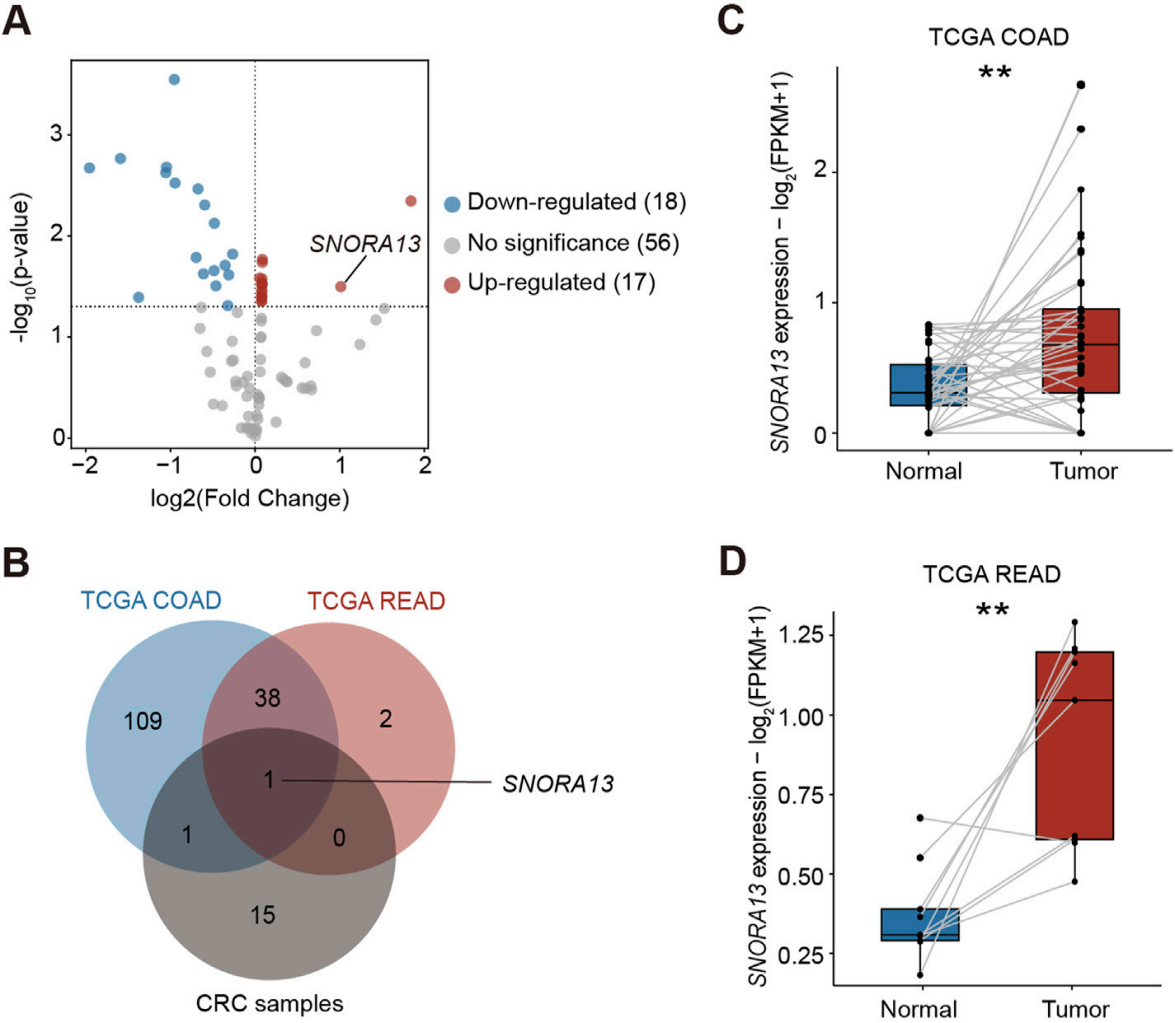
*SNORA13* is upregulated in CRC. **(A)** The volcano plot showed the differentially expressed snoRNAs analyzed by the small RNA array in CRC samples. Red and blue dots represent the upregulated and downregulated snoRNAs, respectively (p < 0.05). *SNORA13* is highlighted in red text. **(B)** A Venn diagram of upregulated snoRNAs in COAD and READ datasets from TCGA and CRC tissues from clinical samples, in which only *SNORA13* overlapped. **(C)** The *SNORA13* expression in paired COAD tumor and adjacent non-tumor tissues from TCGA database. **(D)** The *SNORA13* expression in paired READ tumor and adjacent non-tumor tissues from TCGA database. Data are shown as mean ± S.D., **(C,D)** used paired *t*-test to perform the statistical significance, three biological replicates for each sample were performed, **p < 0.01.

To investigate differential expression of snoRNAs in CRC, we acquired CRC tumor tissues and adjacent non-tumor tissues from patients and conducted a small RNA array analysis to reveal the levels of snoRNAs (Supplementary Table S1). Our results indicated that 17 snoRNAs are upregulated in CRC tissues, among which *SNORA13* and *SNORA65* were highly upregulated (Figure 1A). Next, we analyzed the upregulated snoRNAs in tumor tissues from colon adenocarcinoma (COAD), rectum adenocarcinoma (READ), and corresponding para-tumor tissues from The Cancer Genome Atlas (TCGA) (Supplementary Figures S1A,B) (Weinstein et al., 2013). Our results revealed that *SNORA13* is upregulated in both our clinical CRC samples and databases (Figure 1B). To investigate the upregulation of *SNORA13* in CRC, the expression levels of *SNORA13* in COAD and READ tumor and normal tissues from TCGA database were compared in pairwise, and the statistical examination revealed that *SNORA13* is significantly elevated in both cancer tissues (Figures 1C,D). Next, we examined the *SNORA13* expression levels in CRC cell lines including HT29, HCT116, RKO *versus* normal human intestinal epithelial cell line NCM460, and our data revealed that SNORA13 is significantly upregulated in CRC cell lines (Supplementary Figures S1C).

### Knockdown of *SNORA13* suppresses cell proliferation and growth of CRC

**FIGURE 2:**
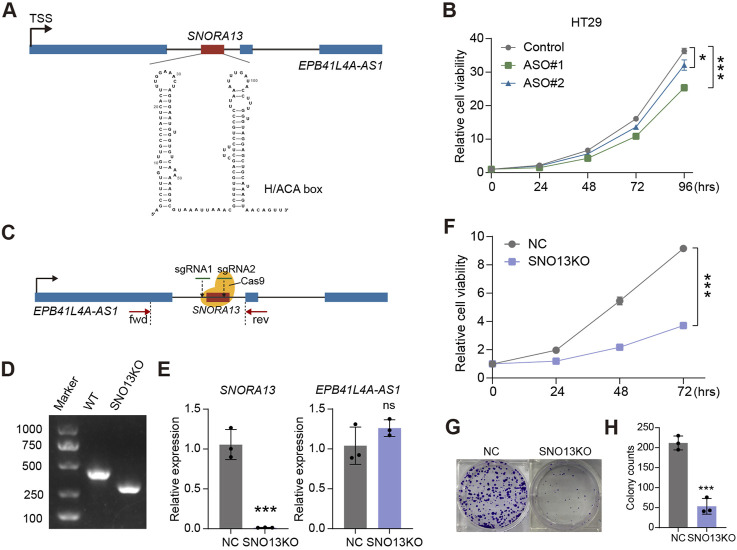
*SNORA13* promotes CRC proliferation *in vitro*. **(A)** Schematic representation of human *SNORA13* which showed the genomic location and second structure of *SNORA13*. **(B)** CCK-8 analysis of the proliferation of HT29 cells upon the indicated transfections. **(C)** Schematic of CRISPR Cas9 based *SNORA13* knockout strategy. fwd and rev refer to the location of forward primer and reverse primer for PCR detection. **(D)** The genotyping agarose gel result for *SNORA13* knockout. The detection region was denoted in **(C)**. **(E)** The relative expression of *SNORA13* and host gene *EPB41L4A-AS1* were determined using RT-qPCR. **(F)** CCK-8 analysis of the proliferation of HT29 NC and SNO13KO cells. **(G,H)** Colony formation analysis in HT29 cells upon *SNORA13* knockout vs the negative control. Unpaired *t*-test was used to perform the statistical significance, three biological replicates for each sample were performed, ns, no significance; *p < 0.05; ***p < 0.001.

SNORA13 localizes in the first intron of *EPB41L4A-AS1*, is a 133 nt length snoRNA containing conserved H/ACA box (Figure 2A) (Kiss et al., 2004). To investigate the role of *SNORA13* in CRC, we performed a loss-of-function (LOF) study by suppressing *SNORA13* levels with antisense oligonucleotides (ASOs). We designed and synthesized 2 ASOs, ASO#1 and ASO#2, and validated their suppressive efficiency by real-time qPCR in multiple cell lines. Our results showed that both ASOs effectively suppress *SNORA13* levels, while not influencing the expression level of *EPB41L4A-AS1* (Supplementary Figures S2A–S2D). Next, cell proliferation assays were conducted using *SNORA13* ASOs in HT29 cells and our data indicated that both ASOs suppress cell proliferation of HT29 CRC cell line (Figure 2B). To validate the role of *SNORA13*, CRISPR-Cas9 genome editing was performed to establish a *SNORA13* knockout HT29 cell line (HT29-SNO13KO) with a pair of sgRNAs flanking *SNORA13* (Figure 2C). Our genotyping results indicated the removal of DNA fragment in KO lines (Figure 2D; Supplementary Figure S3), and real-time qPCR data demonstrated the *SNORA13* was successfully knockdown, while the host gene *EPB41L4A-AS1* was not influenced (Figure 2E). Next, cell proliferation and colony formation assays were performed and our results indicated suppressed cell proliferation and less colony formation in HT29-SNO13KO cells (Figures 2F–H). In addition, we have investigated the ectopic expression of *SNORA13 in vitro* (Supplementary Figure S4A), and our results showed that the *SNORA13* overexpression promotes the clone formation in colorectal carcinoma cells (Supplementary Figures S4B,C).

### Reduced xenograft tumor size in *SNORA13* deficiency cells

**FIGURE 3:**
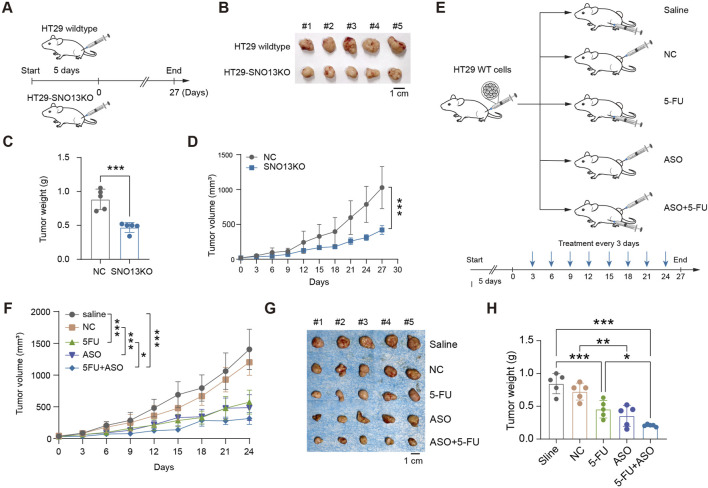
Loss of *SNORA13* inhibits CRC proliferation *in vivo*. **(A)** Schematic diagram of xenografts in *BALB/c* nude mice by inoculating 1 × 10^6^ HT29 NC or HT29-SNO13KO cells. **(B,G)** Representative images of tumors excised on experimental endpoint0. **(C,H)** Tumor weight of each group in nude mice. **(D,F)** Tumor volume measurements of each group were taken on the indicated days. **(E)** Schematic representation of the therapy method. Nude mice were injected subcutaneously with 2 × 10^6^ HT29 wild-type cells and divided into five groups after tumor formation. Each group was treated with saline, NC, 5-FU, ASO, or ASO combined with 5-FU. Data are shown as mean ± S.D., **(C)** used unpaired t-test, (D,F) used two-way ANOVA, and H used one-way ANOVA to perform the statistical significance, n = 5 for each group. *p < 0.05; **p < 0.01; ***p < 0.001.

To study the phenotype of HT29-SNO13KO *in vivo*, xenograft tumor model was established in *BLAB/c* nude mice (Figure 3A). The subcutaneous tumor volume and weight in mice injected with HT29 KO cells were significantly smaller and lower than those in HT29 NC mice (Figures 3B–D). Next, to investigate whether *SNORA13* ASOs could be used to treat CRC tumor therapeutically, a subcutaneous xenograft tumor model was performed, and saline, control ASO (NC), 5-Fluorouracil (5-FU), *SNORA13* ASO (ASO), and combined 5-FU with *SNORA13* ASO were administrated respectively via intratumoral injection twice per week for consecutive 4 weeks (Figure 3E). The tumor size was measured and our data showed that *SNORA13* ASO significantly suppresses tumor volume comparing to control ASO (NC), while combined administration of 5-FU and ASO exhibits superior anti-tumor effect among all groups (Figure 3F). At the endpoint of the experiment, xenografted tumors were isolated and the tumor weight was weighed. Similar to the trend in tumor size, our data indicated decreased tumor weight in ASO and 5-FU + ASO group *versus* controls (Figures 3G,H).

### Knockdown of *SNORA13* suppresses translation efficiency in CRC cells

**FIGURE 4:**
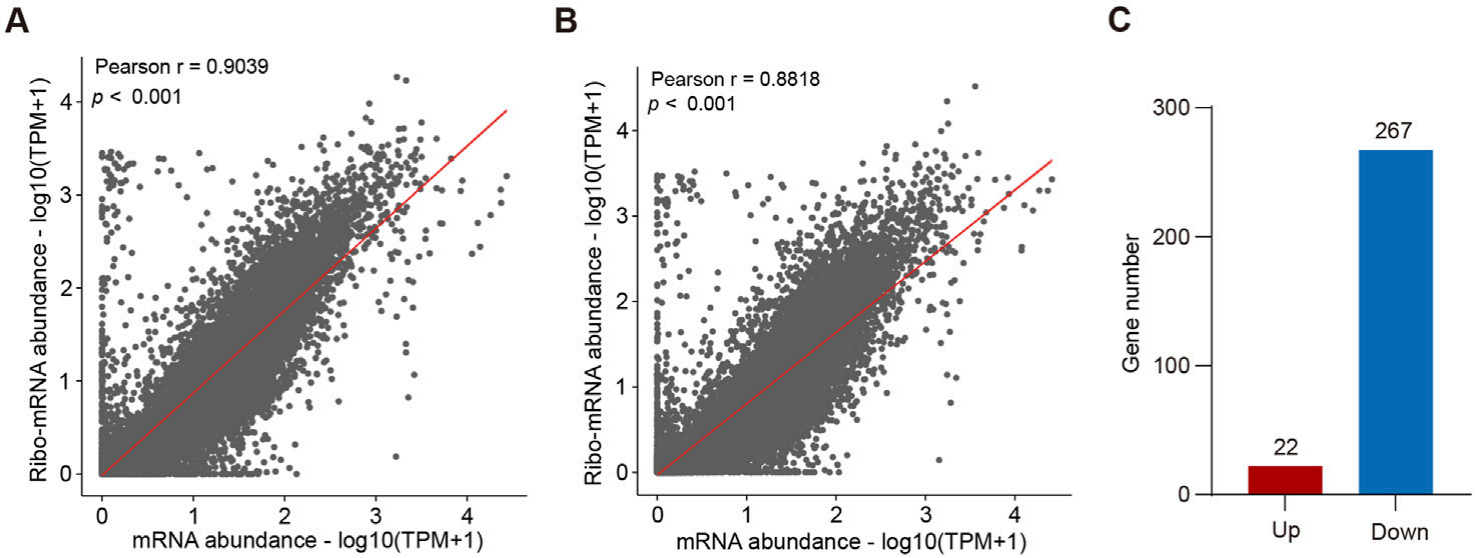
Loss of *SNORA13* reduces protein translation **(A,B)** Scatter plots showing the correlation between mRNA abundance and Ribo-mRNA abundance in HT29 NC **(A)** or HT29-SNO13KO cells **(B)**. **(C)** Bar graph shows the number of proteins with altered translation efficiency after *SNORA13* knockout.

Because snoRNAs are involved in ribosome assembly and protein synthesis, we first performed a puromycin-incorporation assay and found that loss of *SNORA13* significantly inhibited the synthesis of nascent proteins (Supplementary Figures S5A–C). To explore the underlying mechanism of anti-tumor effect upon *SNORA13* knockdown, high throughput RNA sequencing (transcriptome) and Ribo-seq (translatome) were performed using HT29 NC and HT29-SNO13KO cells (Supplementary Tables S2,3). The Ribo-seq analysis showed ribosome occupancy of 598 genes decreased significantly while 341 genes increased significantly (Supplementary Figure S5D). More genes have restricted translation after *SNORA13* knockout. Data analysis revealed that the transcriptome-translatome ratio is greater in HT29 NC cells *versus* HT29-SNO13KO cells, suggesting more mature mRNAs do not participate in protein synthesis upon *SNORA13* knockout (Figures 4A,B). The translation efficiency (TE) altered genes were also analyzed and our data revealed that the TE of 267 genes were downregulated in HT29-SNO13KO compared to HT29 NC cells (Figure 4C). Furthermore, ∼20% of the genes with significantly reduced translation efficiency exhibit a decrease in protein expression levels (Supplementary Figure S5E). Thus, our data indicates that the translation efficiency is suppressed upon deficiency of *SNORA13*, which therefore hampered cell growth and proliferation.

### Knockdown of *SNORA13* represses nicotinamide N-methyltransferase levels

**FIGURE 5:**
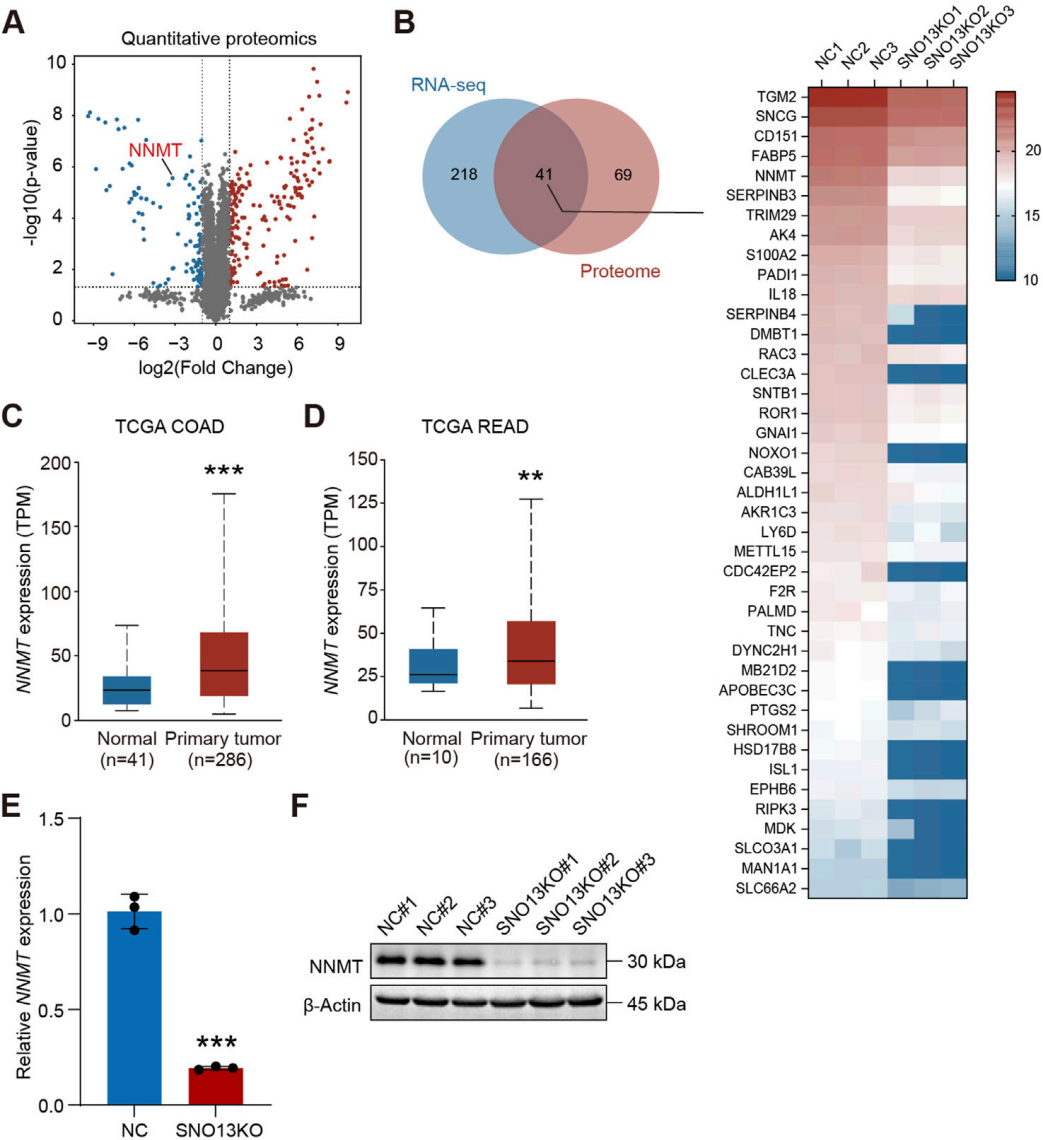
Knockout of *SNORA13* inhibits NNMT expression. **(A)** Volcano plot of differentially expressed proteins upon *SNORA13* silencing. Red and blue dots represent the upregulated and downregulated genes, respectively (*p* < 0.05 and |log_2_fold change|>1). NNMT is highlighted in red text. **(B)** Left panel, Venn diagram was used to identify genes that were co-downregulated in transcriptome and proteome after knockout of *SNORA13*. Right panel, heatmap showed the protein expression levels of the 41 genes screened by the Venn diagram in the proteome between HT29 NC and SNO13KO cells. **(C,D)** The *NNMT* RNA expression in COAD and READ tissues from TCGA database was obtained from UALCAN website. **(E)** The relative expression of *NNMT* in HT29 NC and SNO13KO cells was determined using RT-qPCR. **(F)** NNMT protein levels in HT29 NC and KO cells were determined using Western blotting. Data are shown as mean ± S.D., **(C–E)** used unpaired t-test to perform the statistical significance, three biological replicates for each sample were performed in **(E,F)**, **p < 0.01; ***p < 0.001.

We also performed proteome analysis using HT29 and HT29-SNO13KO cells by 4D label-free quantitative proteomics. Our data revealed that 110 proteins were downregulated, and 197 proteins upregulated upon SNORA13 knockout (log_2_|fold change|>1, p < 0.05) (Figure 5A). Next, we conducted analysis using proteomics and transcriptomics data, and we found 41 common downregulated genes at both RNA and protein levels (Figure 5B). Among these downregulated genes, we noticed that a well-known anti-cancer target, Nicotinamide N-methyltransferase (NNMT), is significantly downregulated upon *SNORA13* knockdown (Supplementary Figure S5F) (Wang et al., 2022). Data re-analysis using TCGA database showed that the levels of NNMT are significantly increased in CRC tissue *versus* adjacent normal tissue (Figures 5C,D) (Song et al., 2020; Yang et al., 2021). To validate, quantitative PCR and Western blotting were performed and our data demonstrated that both RNA and protein levels of NNMT are markedly downregulated upon *SNORA13* deficiency (Figures 5E,F). Together, our data suggested administration of knockdown of *SNORA13* suppresses CRC proliferation and tumor growth via repressing protein synthesis and inhibiting NNMT level.

## Discussion

SnoRNA is a class of nuclear noncoding RNA that plays a crucial role in the modification of ribosomal RNAs and regulation of RNA splicing, thereby modulates protein synthesis (Kim et al., 2019; Rajan et al., 2023). However, the roles of snoRNA in protein synthesis and ribosome heterogenous await further comprehensive investigation (Babaian et al., 2020; Barros-Silva et al., 2021). In this study, our data revealed *SNORA13* is highly expressed in CRC tumor tissues compared to adjacent normal tissues. Moreover, data analysis using TCGA database suggested that the upregulation of *SNORA13* is universal in various types of cancers. Therefore, it justifies the necessity of exploring roles and mechanisms of *SNORA13*. In the context of CRC, we performed loss-of-function study using both transient knockdown of *SNORA13* with ASOs and stable *SNORA13* knockout with genome editing. Both results suggested that losing *SNORA13* suppresses cell proliferation in cultured CRC cell lines and tumor growth in xenograft tumor models. Importantly, our attempts using combinatorial administration of *SNORA13* ASO and 5-FU showed a superior anti-cancer effect compared to 5-FU administration only, suggesting a potential novel therapeutical approach. Mechanistically, we performed transcriptomic (RNA-seq), translatomic (Ribo-seq), and proteomic analyses, which revealed that translation efficiency is markedly reduced upon *SNORA13* knockdown, particularly in cancer cells. Moreover, we found a well-defined target, NNMT, is downregulated upon *SNORA13* knockdown, which impose *SNORA13* ASO as a potential NNMT inhibitor. The understanding of *SNORA13* in modulating NNMT expression needs to be further investigated in future studies.

*SNORA13* is located at the intronic region of *EPB41L4A-AS1*. Previous study reported that *EPB41L4A-AS1* was highly expressed in CRC tissues and plays an important role in promoting cell proliferation, invasion, and migration of CRC (Bin et al., 2021). Since *SNORA13* and *EPB41L4A-AS1* share a common promoter, it is challenging to distinguish the function of *SNORA13* from its host gene *EPB41L4A-AS1*. In this study, we employed a pair of single-guide RNAs (sgRNAs) flanking the *SNORA13* locus to excise the DNA fragment from the genome using CRISPR-Cas9 genome editing (Ran et al., 2013; Zhang S. et al., 2023). Our results demonstrate that this manipulation does not affect the expression of *EPB41L4A-AS1*. Consequently, the suppression of both the host gene *EPB41L4A-AS1* and *SNORA13* may yield a synergistic anti-cancer effect. It is intriguing to investigate whether the genomic removal of the *EPB41L4A-AS1-SNORA13* locus through genome editing could offer enhanced efficacy in the treatment of CRC in future study.

NNMT catalyzes the N-methylation of nicotinamide (NAM) to produce 1-methylnicotinamide (1-MNAM) and S-adenosyl-L-homocysteine (SAH), utilizing a methyl group from S-adenosyl-L-methionine (SAM). This enzymatic activity enables NNMT to play a significant role in reprogramming cellular metabolism (Roberti et al., 2021; Wang et al., 2022). NNMT is highly expressed in various cancer types, which subsequentially alters the epigenetic state of hypomethylated histones and various cancer-related proteins (Ulanovskaya et al., 2013). Inhibition of NNMT leads to a methyl overflow, which enhances trimethylation of histone H3K9 and increases DNA methylation at the promoters of PRDM5 and extracellular matrix-related genes in cancer cells, therefore being regarded as a promising anti-cancer target (Gao et al., 2021; Li et al., 2022; Couto et al., 2023; Sun et al., 2024). However, few studies have addressed the correlation between snoRNAs function and NNMT. Our data demonstrated inhibiting NNMT through modulating snoRNA, providing a potential strategy for developing NNMT inhibitors.

In summary, our data indicates that upregulation of *SNORA13* in CRC and demonstrates its anti-cancer efficacy by suppressing *SNORA13* using ASO. Furthermore, we found that the combined administration of *SNORA13* ASOs and 5-FU offers superior efficacy compared to 5-FU alone, suggesting a promising strategy to enhance 5-FU-based therapy.

## Materials and methods

### Clinical specimens

Clinical CRC samples and paired para-tumor tissues were collected at China-Japan Friendship Hospital. Ethical approval for the study was granted by the ethics committee of China-Japan Friendship Hospital.

### Samples preparation and small RNA array

The surgery removed tumors were washed by 1xPBS twice and flash freezes in liquid nitrogen and transport on dry ice to Aksomics Biotech LLC (Shanghai, China) for small RNA array. In brief, the quality and quantity of total RNA samples were determined by NanoDrop ND-1000 spectrophotometer, and the RNA integrity estimation was analyzed by Bioanalyzer 2100. For each sample, 100 ng of total RNA was dephosphorylated and then denatured by DMSO and enzymatically labeled with Cy3. The labeled RNA was hybridized onto Arraystar Human small RNA Microarray (8 × 15K, Arraystar) and the array was scanned by an Agilent Scanner G2505C. Agilent Feature Extraction software (version 11.0.1.1) was used to analyze acquired array images. Quantile normalization and subsequent data processing were performed using GeneSpring GX v12.1 software package (Agilent Technologies). After normalization, the probe signals having Present (P) or Marginal (M) QC flags in at least 1 out of 10 samples were retained. Multiple probes from the same small RNA were combined into one RNA level. Differentially expressed small RNAs were identified by fold change (FC) with a statistical significance (*p* < 0.05).

### Cell culture

The human CRC cell lines HT29, HCT116, RKO, and the human normal intestinal epithelial cell line NCM460 were purchased from iCell Bioscience (Shanghai, China). HT29 and HCT116 cells were cultured in McCoy’s 5A medium (Procell, Wuhan, China, #PM150710) supplemented with 10% fetal bovine serum (FBS, Yeasen, #40130ES76). RKO cells were cultured in Minimum Essential Medium (MEM, Procell, Wuhan, China, # PM150411) with 10% FBS. NCM460 cells were cultured in Roswell Park Memorial Institute (RPMI) 1640 Medium (Procell, Wuhan, China, #PM150110) with 10% FBS. All cell lines were cultured in a humified incubator, at 37°C and 5% CO_2_.

### Transfections of antisense oligonucleotides (ASOs)

All ASOs (Supplementary Table S4) were synthesized and purchased from RIBOBIO corporation (Guangzhou, China). ASOs were transiently transfected into cultured cells at a final concentration of 50 nM using Lipofectamine 3000 (Invitrogen, United States, #L3000015) following the manufacturer’s guide.

### Puromycin-incorporation assay

Cells were pulsed with complete media only (-puro) or complete media containing 10 μg/mL puromycin (+puro) for 10 min in an incubator, then washed in PBS and chased for 60 min in an incubator. 1 × 10^6^ Cells were fixed in 2% paraformaldehyde in PBS for 15 min at room temperature, washed twice, and then stained in 1:100 Anti-Puromycin antibody clone 12D10, Alexa Fluor 488 Conjugated Antibody (MAEBE343-AF488, Millipore) (+Ab) for 30 min on ice. After washing twice, the cells were used for flow cytometry analysis.

### CRISPR-Cas9 genome editing and *SNORA13* knockout cell line screening

*SNORA13* targeting sgRNAs (sequences provided in Supplementary Table S4) were designed using E-CRISP (http://www.e-crisp.org/E-CRISP/index.html). Lentivirus expressing Cas9 and sgRNAs were purchased from GenePharma corporation (Shanghai, China). In brief, HT29 cells were incubated with the lentivirus and polybrene (Yeasen, #40804ES76) for 48 h. Then the Puromycin (Gibco, #A1113803) was added into the culture supernatant of transfected cells with a final concentration of 2 μg/mL for 24 h. The screening of single clones following methods described in our previous publication (Zhang S. et al., 2023).

### RNA extraction, reverse transcription, and quantitative real-time PCR

The total RNA was lysed in the TRIzol Reagent (Thermo Fisher, #15596026), followed by conventional chloroform extraction. 500 ng of total RNA was reverse transcribed using ReverTra Ace qPCR RT Master Mix with genomic DNA Remover (Toyobo, #FSQ-301). Realtime qPCR was performed using PerfectStart Green qPCR SuperMix (Transgen, #AQ601), and data analysis using *U3* to normalize *SNORA13* expression and *GAPDH* as the reference gene for all other genes. The sequence of all primers used in the study are listed in Supplementary Table S4.

### Cell proliferation assays and colony formation analysis

TransDetect Cell Counting Kit (TransGen, Beijing, China, # FC101) was used for CCK-8 assays to assess cell proliferation. Briefly, cells were seeded into 96-well plates at a density of 3,000 cells per well. At indicated time points, the culture medium was replaced with complete medium containing 10% CCK-8 reagent, and the absorbance was measured at 450 nm 2 h later. For colony formation analysis, cells were plated on 6-well plates at a density of 300 cells per well and cultured for 2 weeks. At the end of the experiment, cells were fixed with 4% paraformaldehyde (Beyotime, China, #P0099) and stained with 0.1% crystal violet for visualization.

### Animal experiments

Four-week-old BALB/c nude mice (male) were purchased from Cyagen Biosciences (Suzhou, China) for animal experiments. Cells washed with PBS were collected and mixed with basement membrane matrix (MCE, #HY-K6001) at a volume ratio of 1:1 before injection. 1 × 10^6^ HT29 *SNORA13* knockout (HT29 KO) or HT29 knockout control (HT29 NC) cells were subcutaneously injected into nude mice flank to establish xenograft models. Tumor growth was monitored every 3 days and tumor volume was calculated using the formula: ab^2^/2, where a = length and b = width. For ASO therapeutic experiments, mice were subcutaneously injected with HT29 wildtype cells on the flank, and tumor volumes were monitored every 3 days. When tumors became palpable, the mice were randomly divided into five subgroups and treated for 4 weeks as follows: 1) saline by intraperitoneal injection every 3 days (control group); 2) 20 mg/kg 5-FU by intraperitoneal injection every 3 days (5-FU treatment group); 3) 5 nmol ASO NC by intratumoral injection every 3 days (ASO control treatment group); 4) 5 nmol ASO2 by intratumoral injection every 3 days (ASO treatment group); or 5) 5 nmol ASO2 + 20 mg/kg 5-FU every 3 days (ASO+5-FU treatment group).

### Western blot analyses

Cells were washed once with PBS in dishes and then lysed on ice for 30 min using protein lysis buffer, which was prepared with RIPA buffer (Beyotime, #P0013B) and 1 mM PMSF (Beyotime, ST507). The lysate was collected and centrifuged at 12,000 rpm for 15 min, at 4°C. After determining protein concentration using the Bradford Protein Assay Kit (Beyotime, #P0006C), proteins were boiled in 5× loading buffer (Beyotime, #P0285) for 10 min and then separated using PAGE gels prepared by Omni-Easy one-step PAGE gel fast preparation kit (Epizyme, #PG222) for 1.5 h at 120 V. They were then transferred onto nitrocellulose membranes (Absin, #abs959) for 2 h at 240 mA, blocked with 5% milk in TBST for 1 h, and then incubated with NNMT (Proteintech, Wuhan, China, #15123-1-AP, 1:2000) or β-Actin (CST, #8H10D10, 1:2000) primary antibodies for 1.5 h. After being washed thrice with TBST, they were incubated with rabbit (SeraCare. #5220-0336, 1:5000) or mouse (SeraCare, #5220-0341, 1:5000) secondary antibodies for 1 h. Finally, the protein signal was visualized on the iBright750 imaging system (Thermo Fisher Scientific).

### RNA-sequencing, Ribo-seq and 4D label-free qualitative proteomics

For RNA-sequencing, total RNA of HT29 NC and HT29 KO cells was isolated using TRIzol reagent according to the manufacturer’s protocol. For Ribo-seq, the cells were digested with trypsin from the culture dish, centrifuged, washed twice with PBS, and finally put into liquid nitrogen for quick freezing. The purification, quality inspection and PCR library construction of samples were completed by GENE DENOVO (Guangzhou, China). For proteomics, the cells were harvested from 10 cm dishes using cell scrapers, washed with PBS and centrifuged to obtain cell pellets, which were then quickly frozen in liquid nitrogen. Proteins were extracted from the cell pellets and 4D label-free qualitative proteomics by OEbiotech Corporation (Shanghai, China).

### Bioinformatics analysis

For differentially expressed snoRNAs analysis, TCGA datasets were downloaded from the UCSC Xena (https://xenabrowser.net/datapages/). Differentially expressed genes were identified using the “DESeq2” package with standard settings in R. Genes with a *p*-value <0.05 and | log_2_ (fold change) | > 2 were considered differentially expressed. All snoRNAs were screened from differentially expressed genes according to the “gene type” classification annotated as “snoRNA” in Ensembl genome browser (https://www.ensembl.org/, Genome assembly: GRCh38.p14). The analyses of NNMT expression in TCGA COAD and READ were performed on the UALCAN website (https://ualcan.path.uab.edu/).

### Statistical analyses

All experiments were done in triplicate. Data analyses were performed using GraphPad Prism version 10. Student’s t-test was used to make statistical comparisons for two groups and one-way analysis of variance (ANOVA) for multiple groups. Cell growth and tumor volume curves were analyzed using two-way ANOVA. Results are presented as the mean ± standard deviation. *p* < 0.05 indicates statistically significant differences. *, **, and *** indicate *p* < 0.05, <0.01, and <0.001, respectively.

## Data Availability

The datasets presented in this study can be found in online repositories. The names of the repository/repositories and accession number(s) can be found in the article/Supplementary Material.

## References

[B1] Babaian A. Rothe K. Girodat D. Minia I. Djondovic S. Milek M. (2020). Loss of m(1)acp(3)ψ ribosomal RNA modification is a major feature of cancer. Cell. Rep. 31, 107611. 10.1016/j.celrep.2020.107611 32375039

[B2] Bachellerie J. P. Cavaillé J. Hüttenhofer A. (2002). The expanding snoRNA world. Biochimie 84, 775–790. 10.1016/s0300-9084(02)01402-5 12457565

[B3] Barros-Silva D. Klavert J. Jenster G. Jerónimo C. Lafontaine D. L. J. Martens-Uzunova E. S. (2021). The role of OncoSnoRNAs and ribosomal RNA 2'-O-methylation in cancer. RNA Biol. 18, 61–74. 10.1080/15476286.2021.1991167 34775914 PMC8677010

[B4] Baßler J. Hurt E. (2019). Eukaryotic ribosome assembly. Annu. Rev. Biochem. 88, 281–306. 10.1146/annurev-biochem-013118-110817 30566372

[B5] Biller L. H. Schrag D. (2021). Diagnosis and treatment of metastatic colorectal cancer: a review. Jama 325, 669–685. 10.1001/jama.2021.0106 33591350

[B6] Bin J. Nie S. Tang Z. Kang A. Fu Z. Hu Y. (2021). Long noncoding RNA EPB41L4A-AS1 functions as an oncogene by regulating the Rho/ROCK pathway in colorectal cancer. J. Cell. Physiol. 236, 523–535. 10.1002/jcp.29880 32557646

[B7] Blondy S. David V. Verdier M. Mathonnet M. Perraud A. Christou N. (2020). 5-Fluorouracil resistance mechanisms in colorectal cancer: from classical pathways to promising processes. Cancer Sci. 111, 3142–3154. 10.1111/cas.14532 32536012 PMC7469786

[B8] Bortolin-Cavaillé M. L. Quillien A. Thalalla Gamage S. Thomas J. M. Sas-Chen A. Sharma S. (2022). Probing small ribosomal subunit RNA helix 45 acetylation across eukaryotic evolution. Nucleic Acids Res. 50, 6284–6299. 10.1093/nar/gkac404 35648437 PMC9226516

[B9] Bray F. Laversanne M. Sung H. Ferlay J. Siegel R. L. Soerjomataram I. (2024). Global cancer statistics 2022: GLOBOCAN estimates of incidence and mortality worldwide for 36 cancers in 185 countries. CA Cancer J. Clin. 74, 229–263. 10.3322/caac.21834 38572751

[B10] Couto J. P. Vulin M. Jehanno C. Coissieux M. M. Hamelin B. Schmidt A. (2023). Nicotinamide N-methyltransferase sustains a core epigenetic program that promotes metastatic colonization in breast cancer. Embo J. 42, e112559. 10.15252/embj.2022112559 37259596 PMC10308372

[B11] Dekker E. Tanis P. J. Vleugels J. L. A. Kasi P. M. Wallace M. B. (2019). Colorectal cancer. Lancet 394, 1467–1480. 10.1016/S0140-6736(19)32319-0 31631858

[B12] Ender C. Krek A. Friedländer M. R. Beitzinger M. Weinmann L. Chen W. (2008). A human snoRNA with microRNA-like functions. Mol. Cell. 32, 519–528. 10.1016/j.molcel.2008.10.017 19026782

[B13] Gao Y. Martin N. I. Van Haren M. J. (2021). Nicotinamide N-methyl transferase (NNMT): an emerging therapeutic target. Drug Discov. Today 26, 2699–2706. 10.1016/j.drudis.2021.05.011 34029690

[B14] Huang Z. H. Du Y. P. Wen J. T. Lu B. F. Zhao Y. (2022). snoRNAs: functions and mechanisms in biological processes, and roles in tumor pathophysiology. Cell. Death Discov. 8, 259. 10.1038/s41420-022-01056-8 35552378 PMC9098889

[B15] Kim D. S. Camacho C. V. Nagari A. Malladi V. S. Challa S. Kraus W. L. (2019). Activation of PARP-1 by snoRNAs controls ribosome biogenesis and cell growth via the RNA helicase DDX21. Mol. Cell. 75, 1270–1285. 10.1016/j.molcel.2019.06.020 31351877 PMC6754283

[B16] Kishore S. Stamm S. (2006). The snoRNA HBII-52 regulates alternative splicing of the serotonin receptor 2C. Science 311, 230–232. 10.1126/science.1118265 16357227

[B17] Kiss A. M. Jády B. E. Bertrand E. Kiss T. (2004). Human box H/ACA pseudouridylation guide RNA machinery. Mol. Cell. Biol. 24, 5797–5807. 10.1128/MCB.24.13.5797-5807.2004 15199136 PMC480876

[B18] Kiss T. (2002). Small nucleolar RNAs: an abundant group of noncoding RNAs with diverse cellular functions. Cell. 109, 145–148. 10.1016/s0092-8674(02)00718-3 12007400

[B19] Li X. Y. Pi Y. N. Chen Y. Zhu Q. Xia B. R. (2022). Nicotinamide N-methyltransferase: a promising biomarker and target for human cancer therapy. Front. Oncol. 12, 894744. 10.3389/fonc.2022.894744 35756670 PMC9218565

[B20] Longley D. B. Harkin D. P. Johnston P. G. (2003). 5-fluorouracil: mechanisms of action and clinical strategies. Nat. Rev. Cancer 3, 330–338. 10.1038/nrc1074 12724731

[B21] Mcmahon M. Contreras A. Ruggero D. (2015). Small RNAs with big implications: new insights into H/ACA snoRNA function and their role in human disease. Wiley Interdiscip. Rev. RNA 6, 173–189. 10.1002/wrna.1266 25363811 PMC4390053

[B22] Rajan K. S. Madmoni H. Bashan A. Taoka M. Aryal S. Nobe Y. (2023). A single pseudouridine on rRNA regulates ribosome structure and function in the mammalian parasite Trypanosoma brucei. Nat. Commun. 14, 7462. 10.1038/s41467-023-43263-6 37985661 PMC10662448

[B23] Ran F. A. Hsu P. D. Wright J. Agarwala V. Scott D. A. Zhang F. (2013). Genome engineering using the CRISPR-Cas9 system. Nat. Protoc. 8, 2281–2308. 10.1038/nprot.2013.143 24157548 PMC3969860

[B24] Roberti A. Fernández A. F. Fraga M. F. (2021). Nicotinamide N-methyltransferase: at the crossroads between cellular metabolism and epigenetic regulation. Mol. Metab. 45, 101165. 10.1016/j.molmet.2021.101165 33453420 PMC7868988

[B25] Sethy C. Kundu C. N. (2021). 5-Fluorouracil (5-FU) resistance and the new strategy to enhance the sensitivity against cancer: implication of DNA repair inhibition. Biomed. Pharmacother. 137, 111285. 10.1016/j.biopha.2021.111285 33485118

[B26] Shin A. E. Giancotti F. G. Rustgi A. K. (2023). Metastatic colorectal cancer: mechanisms and emerging therapeutics. Trends Pharmacol. Sci. 44, 222–236. 10.1016/j.tips.2023.01.003 36828759 PMC10365888

[B27] Song M. Li Y. Miao M. Zhang F. Yuan H. Cao F. (2020). High stromal nicotinamide N-methyltransferase (NNMT) indicates poor prognosis in colorectal cancer. Cancer Med. 9, 2030–2038. 10.1002/cam4.2890 31989785 PMC7064029

[B28] Sun W. D. Zhu X. J. Li J. J. Mei Y. Z. Li W. S. Li J. H. (2024). Nicotinamide N-methyltransferase (NNMT): a key enzyme in cancer metabolism and therapeutic target. Int. Immunopharmacol. 142, 113208. 10.1016/j.intimp.2024.113208 39312861

[B29] Ulanovskaya O. A. Zuhl A. M. Cravatt B. F. (2013). NNMT promotes epigenetic remodeling in cancer by creating a metabolic methylation sink. Nat. Chem. Biol. 9, 300–306. 10.1038/nchembio.1204 23455543 PMC3631284

[B30] Wang W. Yang C. Wang T. Deng H. (2022). Complex roles of nicotinamide N-methyltransferase in cancer progression. Cell. Death Dis. 13, 267. 10.1038/s41419-022-04713-z 35338115 PMC8956669

[B31] Weinstein J. N. Collisson E. A. Mills G. B. Shaw K. R. Ozenberger B. A. Ellrott K. (2013). The cancer genome Atlas pan-cancer analysis project. Nat. Genet. 45, 1113–1120. 10.1038/ng.2764 24071849 PMC3919969

[B32] Williams G. T. Farzaneh F. (2012). Are snoRNAs and snoRNA host genes new players in cancer? Nat. Rev. Cancer 12, 84–88. 10.1038/nrc3195 22257949

[B33] Yang J. Tong Q. Zhang Y. Yuan S. Gao Y. Deng K. (2021). Overexpression of Nicotinamide N-methyltransferase mainly covers stroma of colorectal cancer and correlates with unfavorable survival by its product 1-MNA. J. Cancer 12, 6170–6181. 10.7150/jca.56419 34539890 PMC8425209

[B34] Zhang S. Chen Y. Dong K. Zhao Y. Wang Y. Wang S. (2023a). BESST: a novel LncRNA knockout strategy with less genome perturbance. Nucleic Acids Res. 51, e49. 10.1093/nar/gkad197 36938886 PMC10201427

[B35] Zhang X. Wang C. Xia S. Xiao F. Peng J. Gao Y. (2023b). The emerging role of snoRNAs in human disease. Genes. Dis. 10, 2064–2081. 10.1016/j.gendis.2022.11.018 37492704 PMC10363644

