# Supplementary figures and images for: SNORA13 antisense oligonucleotides enhances the therapeutical effects of 5-fluorouracil in colon adenocarcinoma
Source: Front Pharmacol. 2025 Jun 2;16:1564682. doi: 10.3389/fphar.2025.1564682 (PMC12171199; doi:10.3389/fphar.2025.1564682)

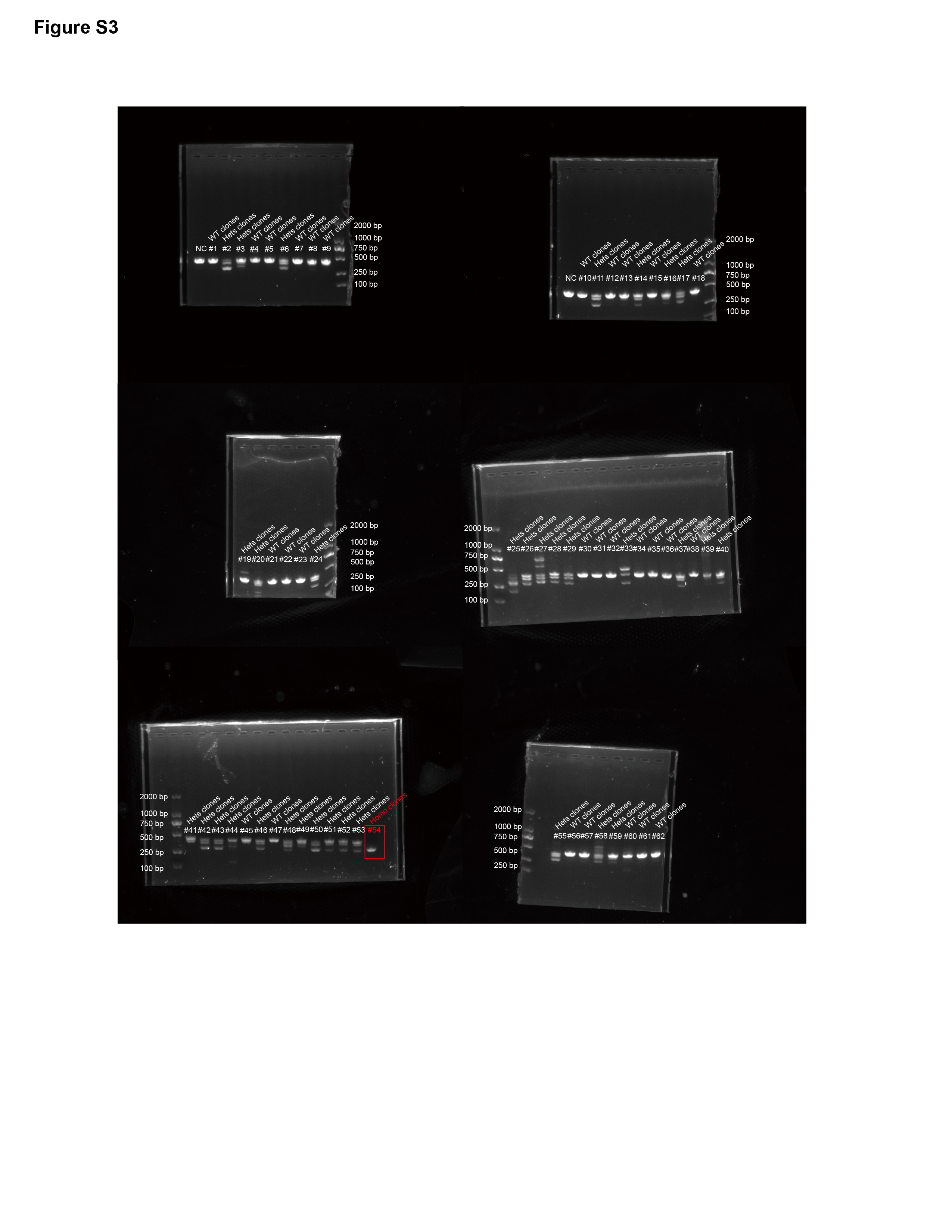

Supplement: Supplementary file 2 [file Image3.jpeg]

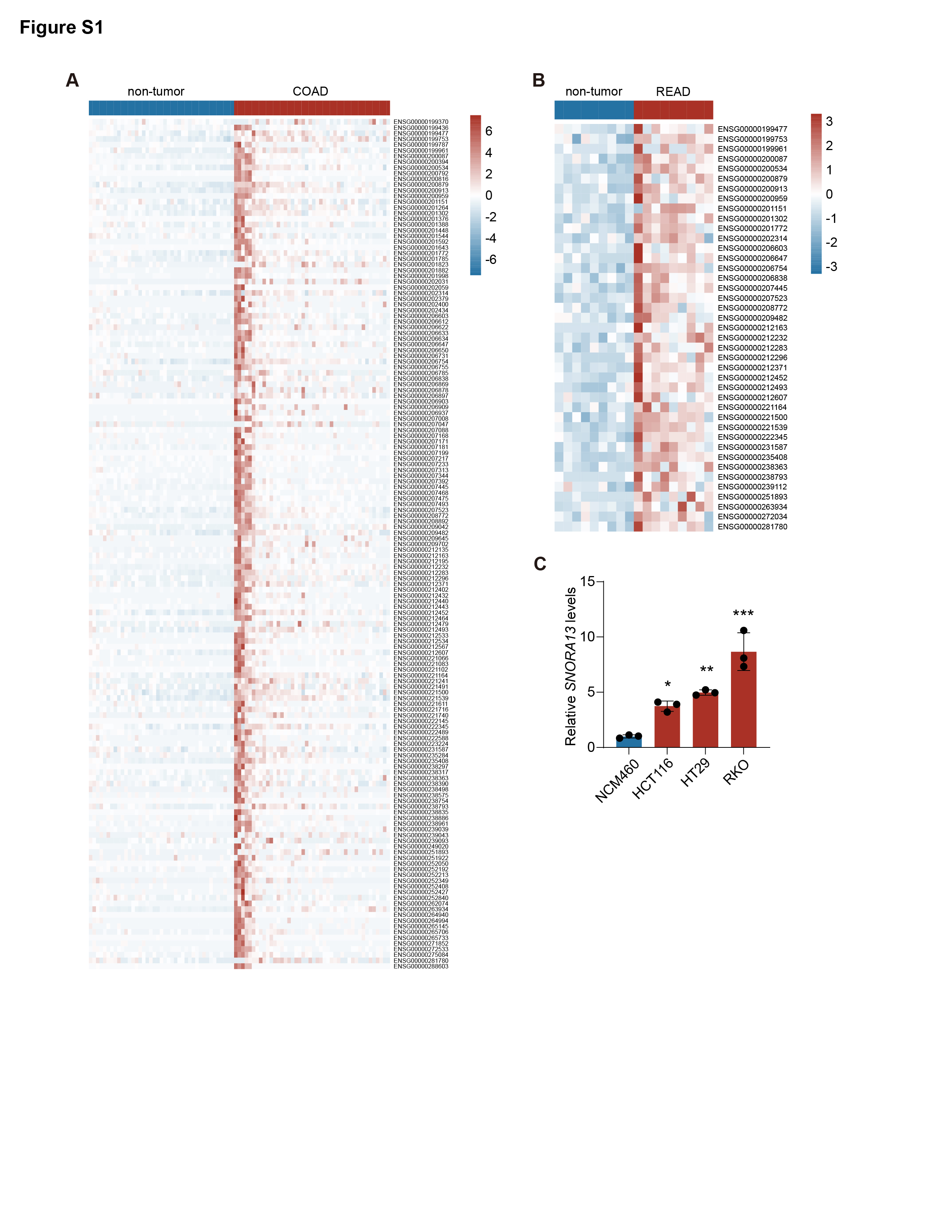

Supplement: Supplementary file 4 [file Image1.jpeg]

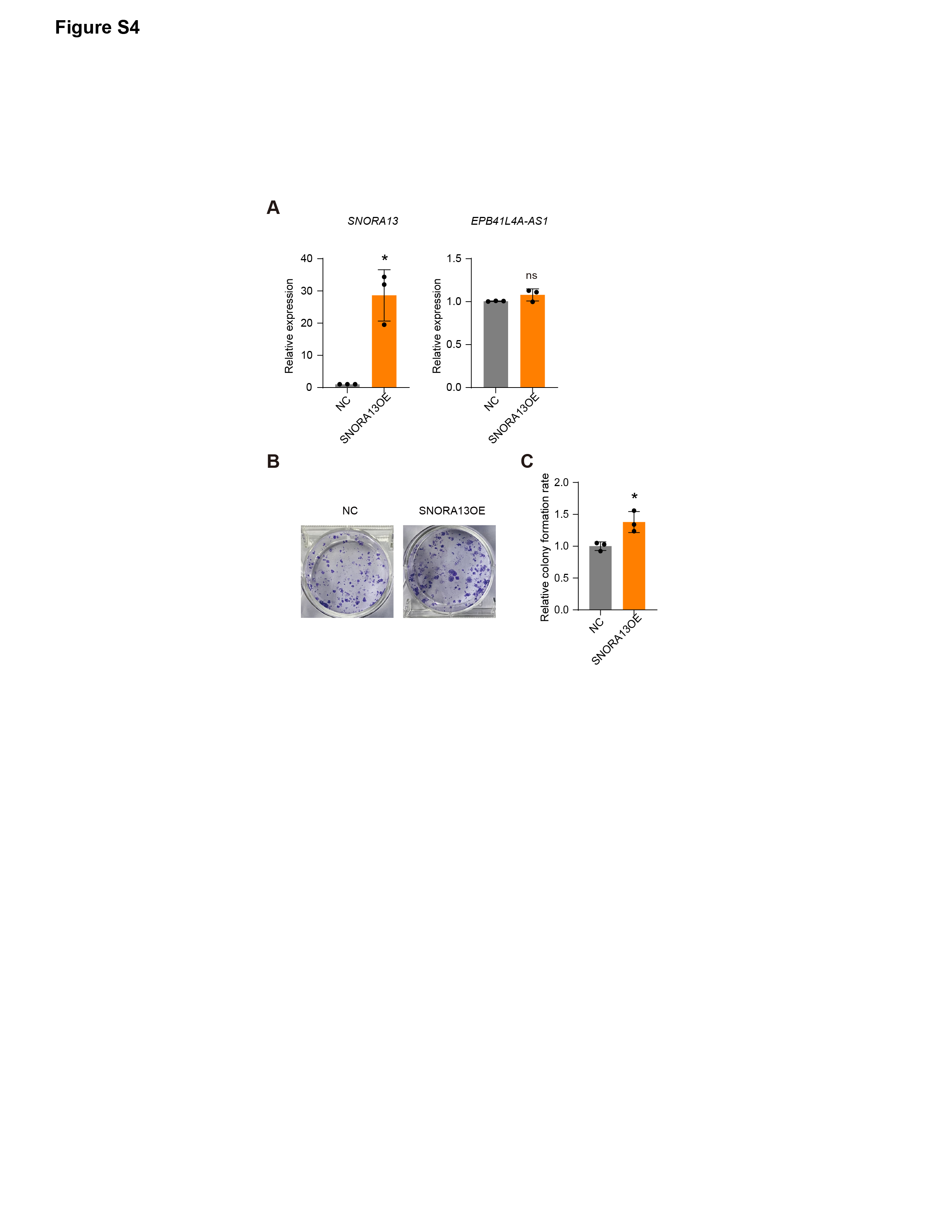

Supplement: Supplementary file 5 [file Image4.jpeg]

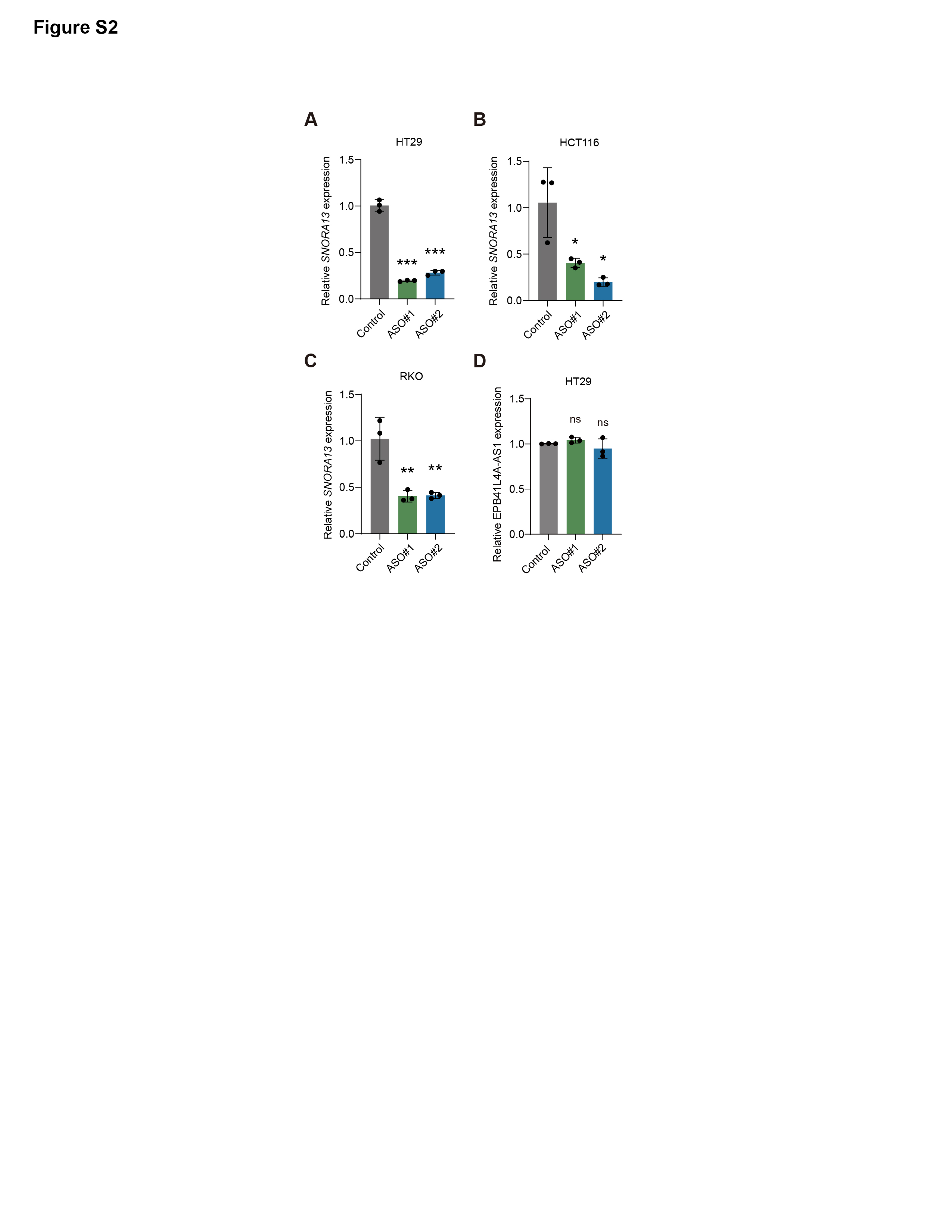

Supplement: Supplementary file 6 [file Image2.jpeg]

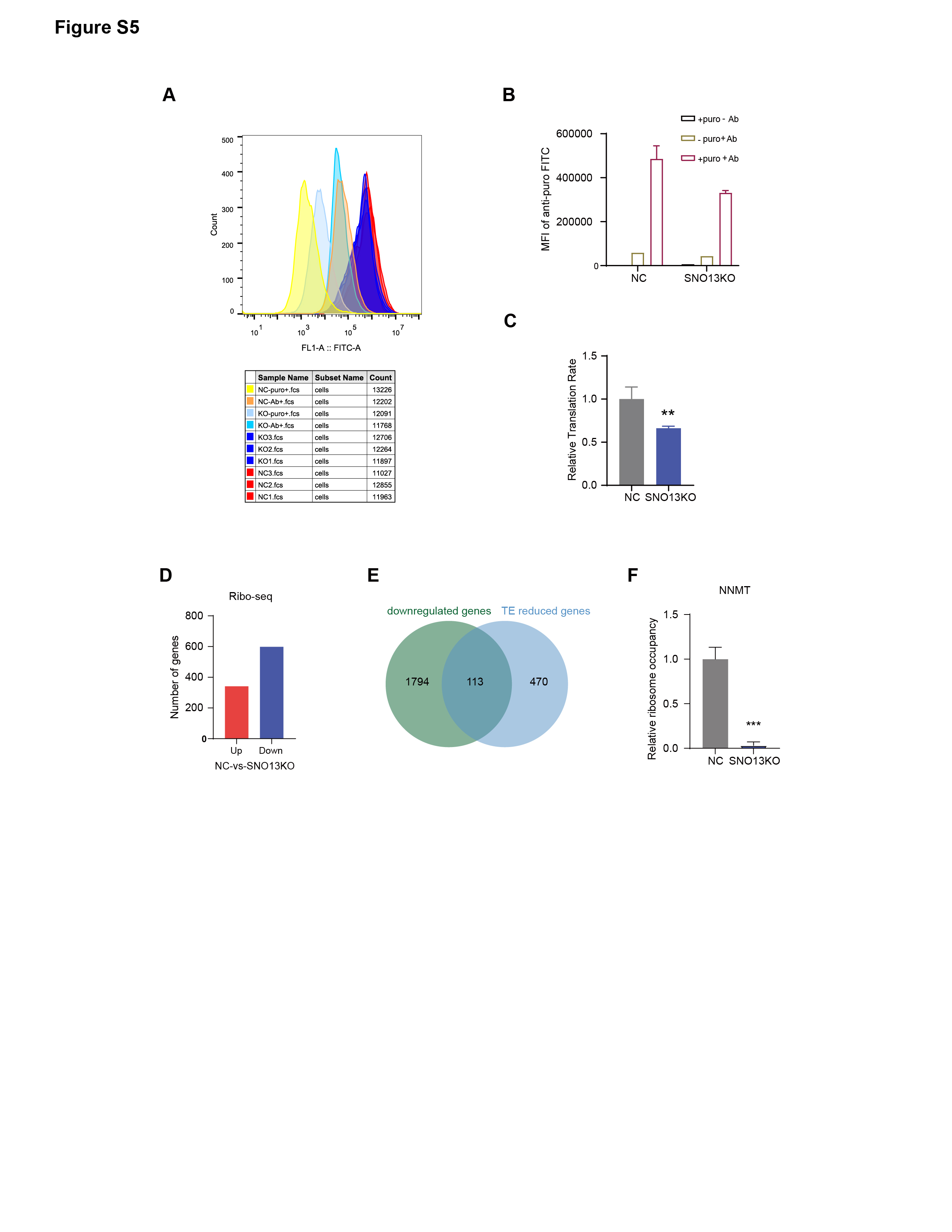

Supplement: Supplementary file 7 [file Image5.jpeg]
