# Supplementary material for: SNORA13 antisense oligonucleotides enhances the therapeutical effects of 5-fluorouracil in colon adenocarcinoma
Source: Front Pharmacol. 2025 Jun 2;16:1564682. doi: 10.3389/fphar.2025.1564682 (PMC12171199; doi:10.3389/fphar.2025.1564682)
Supplement: Supplementary file 9 [file Table4.docx]

| **Oligonucleotide name/description** | **Sequence (5' - 3')** | **Comments** |
| --- | --- | --- |
| SNORA13_sgRNA1 | GAGGTAGGCTTTATGGCATA | sgRNAs used to generate SNORA13 KO cell lines |
| SNORA13_sgRNA2 | GCCCTTAAATTTGATACCTT |  |
| ASO1 | TCCTACACCAAAGGTATCAA | ASOs for knockdown SNORA13 |
| ASO2 | GCAAAGCGTTTAATTTACGC |  |
| KO_SNORA13 PCR fwd primer | GGGGAGCTTCGTCGATTTGT | Primers used to detect genomic knockout |
| KO_SNORA13 PCR rev primer | CGCGTGGAAACGTAAAAGCA |  |
| SNORA13 qRT-PCR fwd primer | AGCCTTTGTGTTGCCCATT | Primers used for qPCR |
| SNORA13 qRT-PCR rev primer | GCAGCTCCTACACCAAAGGTAT |  |
| GAPDH qRT-PCR fwd primer | AAGGCTGTGGGCAAGG |  |
| GAPDH qRT-PCR rev primer | TGGAGGAGTGGGTGTCG |  |
| NNMT qRT-PCR fwd primer | ATATTCTGCCTAGACGGTGTGA |  |
| NNMT qRT-PCR rev primer | TCAGTGACGACGATCTCCTTAAA |  |
| EPB41L4A-AS1 qRT-PCR fwd primer | CCTGGTTTTATTTTCGTCA |  |
| EPB41L4A-AS1 qRT-PCR rev primer | ATCCATCTTCCACCTGTAG |  |
| U3 qRT-PCR fwd primer | CCACGAGGAAGAGAGGTAGC |  |
| U3 qRT-PCR rev primer | CACTCAGACCGCGTTCTCTC |  |

**Table S4: Oligonucleotide sequences**
